# Gene set mediation analysis in high-dimensional epigenetic studies

**DOI:** 10.1093/bib/bbag510

**Published:** 2026-09-29

**Authors:** Yuzhao Gao, Nan Qiao, Lin Yang, Yan Liu, Ruiling Fang, Ningning Shen, Yao Zhang, Gang Wang, Yaoping Li, Yuehua Cui

**Affiliations:** Department of Applied Statistics, School of Statistics, Shanxi University of Finance and Economics, No. 140 Wucheng Road, Taiyuan 030006, China; Office of Cancer Prevention and Control, Shanxi Province Cancer Hospital/Shanxi Hospital Affiliated to Cancer Hospital, Chinese Academy of Medical Sciences/Cancer Hospital Affiliated to Shanxi Medical University, No. 3 Zhigongxin Street, Taiyuan 030013, China; Department of Applied Statistics, School of Statistics, Shanxi University of Finance and Economics, No. 140 Wucheng Road, Taiyuan 030006, China; Office of Cancer Prevention and Control, Shanxi Province Cancer Hospital/Shanxi Hospital Affiliated to Cancer Hospital, Chinese Academy of Medical Sciences/Cancer Hospital Affiliated to Shanxi Medical University, No. 3 Zhigongxin Street, Taiyuan 030013, China; Division of Health Statistics, School of Public Health, Shanxi Medical University, No. 56 Xinjian South Road, Taiyuan 030001, China; Division of Health Statistics, School of Public Health, Shanxi Medical University, No. 56 Xinjian South Road, Taiyuan 030001, China; School of Health Service and Management, Shanxi University of Chinese Medicine, No. 121 Daxue Street, Jinzhong 030619, China; Department of Medical Equipment, Shanxi Cardiovascular Hospital, No. 18 Yifen Street, Taiyuan 030024, China; Department of Colorectal & Anal Surgery, Affiliated Provincial Hospital of Shanxi Medical University, No. 29 Shangtasi Street, Taiyuan 030012, China; Department of Statistics and Probability, Michigan State University, Wells Hall, 619 Red Cedar Road, East Lansing, MI 48824, United States

**Keywords:** DNA methylation, functional principal component analysis, high-dimensional mediation model, *P*-value combination

## Abstract

Mediation analysis has significantly enhanced the elucidation of underlying epigenetic mechanisms by identifying mediators that influence the effect of an exposure on an outcome. In epigenomics research, the exponential increase in the dimensionality of mediators necessitates the development of advanced high-dimensional mediation models. Here we introduce a novel Gene Set High-Dimensional Mediation Analysis (gHDMA) model to understand the gene-level mediation role of DNA methylations, building upon our previous HDMA method. We begin our analysis by applying functional principal component analysis to capture gene variation signals across regions, thereby enhancing the model's statistical power. Following this, we employ the Sure Independence Screening method, based on Wilks' *\varLambda*-test, to efficiently reduce the dimensionality to a more manageable level, enabling high-dimensional inference. Our model is distinguished by its minimal reliance on stringent assumptions about the relationship between mediators and the outcome. Extensive simulation studies, by varying the type of basis function, the nature of the outcome variable, and the dimensionality of the gene region, demonstrate the robust performance of the gHDMA model. A corroborative case study further validates the methodology's efficacy. The gHDMA model emerges as a powerful analytical tool, with an enhanced capacity to identify genes characterized by hypermethylation or hypomethylation that mediate the effects of exposure on biological outcomes.

## Introduction

Mediation analysis provides a statistical framework for elucidating the mechanisms through which an exposure variable *(X)* exerts its influence on an outcome variable *(Y)* via one or more intermediate variables, known as mediators *(M)*. The primary objective is to identify potential mediators and to quantify the extent to which the total effect of *X* on *Y* is transmitted through these mediators. Understanding such mediation mechanisms can reveal key biological pathways and inform the development of targeted interventions across diverse scientific domains.

Classical mediation analysis assumes a causal pathway of the form *X*→*M*→*Y* [1], where *X* represents an environmental exposure and *Y* represents a disease outcome in epidemiological applications. This chain encapsulates how the mediator *M* transmits the influence of the exposure on disease onset. In epigenetic studies, DNA methylation (DNAm) is one of the most extensively studied epigenetic mechanisms regulating gene expression without altering the underlying DNA sequence. By influencing chromatin accessibility and transcriptional activity, DNAm plays essential roles in embryonic development, cellular differentiation, genomic stability, and tissue-specific gene regulation. Increasing evidence suggests that environmental exposures are associated with alterations in DNAm patterns (*X*→*M*), which, in turn, contribute to disease development (*M*→*Y*) [2, 3]. Conducting mediation analysis on DNAm data thus enables the construction of mechanistic chains of the form environmental exposure → DNAm pattern → disease outcome. Investigating these changes deepens our understanding of molecular and epigenetic pathways linking the environment to disease risk, thereby advancing our knowledge of the intricate interplay between environmental factors, the genome, and disease pathogenesis.

Aberrant DNAm patterns, including gene hypermethylation and hypomethylation, have been implicated in numerous complex diseases through their effects on gene transcriptional regulation. Although such changes do not alter the DNA sequence itself, they can substantially influence gene transcriptional activity, thereby contributing to disease onset. Consequently, the study of DNAm alterations has become central to large-scale analyses of genome-wide methylation data. Despite its biological importance, elucidating the functional role of DNAm remains challenging because genome-wide methylation data are characterized by extremely high dimensionality, strong correlations among neighboring CpG sites, heterogeneous methylation patterns across genomic regions, and substantial inter-individual variability. Together, these features hinder mediator discovery and demand robust statistical methods tailored to high-dimensional methylation data.

Early epigenetic mediation studies primarily focused on single-site analyses, testing each CpG site independently to evaluate whether its methylation level mediates the relationship between an exposure and an outcome [4–6]. While single-site mediation analysis has been widely applied to identify potential CpG mediators, it faces several limitations. First, the biological interpretability of individual CpG sites is limited, as genes, rather than isolated CpGs, represent the functional units of inheritance and regulation. Second, DNAm levels at individual sites can be highly variable across populations, leading to instability and poor reproducibility of single-site findings. Third, single-site models fail to account for the correlation structure among neighboring CpG sites, which often exhibit strong spatial and functional dependencies. Fourth, testing tens of thousands of CpGs individually introduces severe multiple testing burdens and low statistical power to detect subtle but biologically meaningful effects.

To overcome these challenges, recent studies have extended mediation analysis to multivariate and gene-based frameworks. Several methods have been proposed to model multiple mediators jointly or to perform variable selection among correlated mediators. For instance, lasso-based mediation models (e.g. Ye *et al.* [7]) apply regularization to select important CpGs from high-dimensional DNAm data. Group lasso formulations (e.g. Jérolon *et al.* [8]) incorporate group structures (e.g. CpGs within genes) to improve interpretability. Kernel-based approaches (e.g. Fang *et al.* [9]) have also been proposed to capture nonlinear effects and complex mediation structures. Despite these advances, several challenges remain. First, many variable-selection or penalized regression approaches treat CpG sites as independent features, neglecting their spatial continuity and correlation within genes. Furthermore, kernel and group-lasso methods often require dense and complete data, making them sensitive to missing CpGs or inconsistent site coverage across studies. In addition, these approaches typically aggregate site-level information using linear combinations or average signals, which may not adequately capture nonlinear methylation patterns within gene regions. Finally, although group-Lasso approaches such as Jérolon *et al.* [8] can accommodate high-dimensional mediation settings, they remain primarily site-level selection procedures, treating individual CpGs as candidate mediators rather than modeling coordinated methylation patterns across CpGs within a gene. Consequently, they are more suited to identifying individual CpG mediators than to quantifying gene-level mediation driven by jointly varying CpG profiles.

To address these challenges, we propose a gene set high-dimensional mediation analysis framework (gHDMA) for identifying gene-level DNAm mediators in high-dimensional epigenetic studies. The proposed framework integrates functional principal component analysis (FPCA) with high-dimensional mediation inference to transform correlated CpG methylation profiles within each gene into low-dimensional gene-level representations modeled as smooth functions [10], while preserving the major regional methylation patterns. Compared with existing approaches, gHDMA improves biological interpretability through gene-level mediation inference, accommodates the spatial correlation among neighboring CpG sites via functional modeling, and enhances computational scalability for large-scale methylation studies. Extensive simulation studies and a real DNAm application demonstrate that the proposed framework achieves accurate, computationally efficient, and biologically interpretable gene-level mediation inference, providing an effective tool for investigating epigenetic mechanisms linking environmental exposures to complex diseases.

## Statistical method

The proposed framework aims to identify gene-level mediators that transmit the effect of an exposure variable *(X)* on an outcome variable *(Y)* through changes in DNAm. The use of gene-level DNAm data substantially reduces dimensionality compared to site-level analyses; however, the number of genes typically still far exceeds the available sample size. When each gene is considered as a potential mediator, addressing the high dimensionality of gene-level DNAm data becomes essential to ensure both computational feasibility and statistical interpretability.

Building upon the foundation of our previous work [4], this model retains the classical mediation structure originally introduced by Baron and Kenny [11], while extending it to accommodate high-dimensional mediators derived from gene-level DNAm features. The methodological design integrates functional data representation, dimensionality reduction, and sparse estimation to model complex mediation pathways in high-dimensional omics data. The overall structure of the mediation model, including the relationships among the exposure, mediator, and outcome variables, is illustrated schematically in Fig. 1.

**Figure 1:**
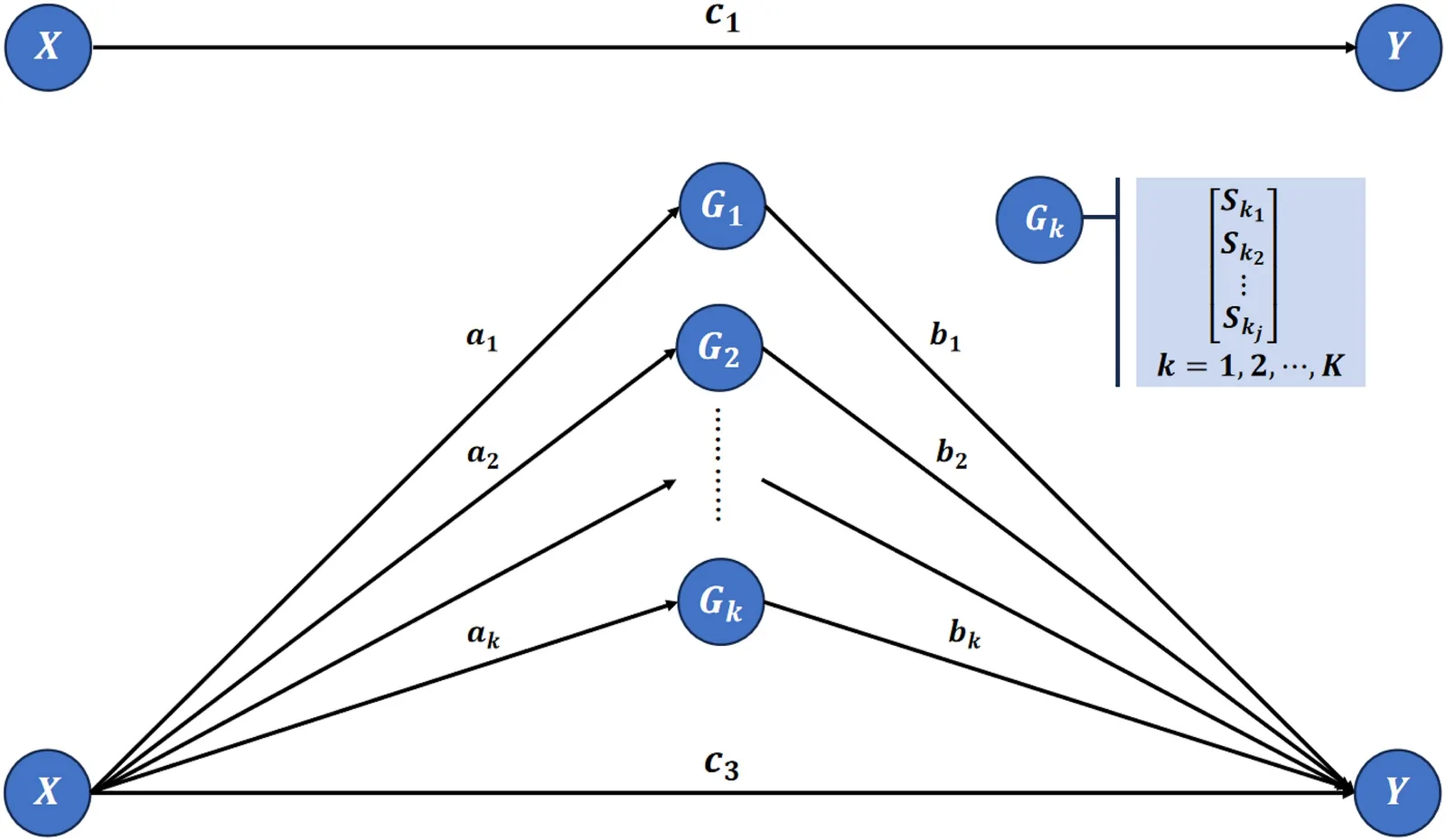
The gene-based mediation model is illustrated in the path diagram, which depicts the total and direct effects of exposure *(X)* on the outcome *(Y)*, as well as the indirect effect of exposure on outcome mediated by the gene mediator *(G)*. Single arrows denote the hypothesized causal pathways, where ${a}_k$ is the effect of *X* on mediator ${G}_k$, ${b}_k$ is the effect of ${G}_k$ on *Y*, and ${c}_3$ is the direct effect of *X* on *Y*. The total effect $\left({c}_1\right)$ is decomposed into the direct effect and the indirect effect.

Let *X* represent an exposure variable, ${G}_i,i=1,2,\cdots, k$ denote the $i\textrm{th}$ gene containing sites ${m}_i$, with *Y* denoting an outcome variable. Figure 1 illustrates the mediation analysis model with gene-based mediators. Mediators within the same gene region are typically correlated, and properly accounting for this correlation can enhance the power to detect gene-based mediation effects, regardless of whether the genes exhibit minor or major effects. This gene-based mediation analysis framework therefore motivates the development of a model capable of identifying genes that mediate the effects of an exposure on an outcome.

Figure 1 presents the gene-based high-dimensional mediation model employed in this study, in which mediators are represented by genes rather than individual CpG sites. Compared with the model proposed in our previous study [4], the present high-dimensional mediation framework aligns more closely with biological interpretability and gene-level inference. The structure of the model is defined as follows:


(1) \begin{eqnarray*} \left\{\begin{array}{@{}c}Y={\theta}_1+{c}_1X+{e}_1\kern9.5em \\{}{G}_i={\theta}_{2i}+{a}_iX+{e}_{2i},i=1,\dots, k.\kern3em \\{}Y={\theta}_3+{c}_3X+{\sum}_{i=1}^k{b}_i{G}_i+{e}_3\kern3.75em \end{array}\right. \end{eqnarray*}

where ${G}_i,i=1,\dots, k$ represents the $i\mathrm{th}$ mediator variable corresponding to the $i\mathrm{th}$ gene region. The parameter ${c}_1$ denotes the total effect of the exposure variable *X* on the outcome variable *Y*, whereas ${c}_3$ represents the direct effect of *X* on *Y*, adjusted for the effects of all mediators. Model (1) applies to a continuous *Y* variable and the total mediation effect can be calculated as ${c}_1-{c}_3$. For a categorical *Y* variable, taking the mathematical expectation of both sides of Model (1) yields:


(2) \begin{eqnarray*} \left\{\begin{array}{@{}l}E(Y)={\theta}_1+{c}_1X\kern11.5em \\{}{G}_i={\theta}_{2i}+{a}_iX+{e}_{2i},i=1,\dots, k.\kern3.75em \\{}E(Y)={\theta}_3+{c}_3X+{\sum}_{i=1}^k{b}_i{G}_i\kern5.25em \end{array}\right. \end{eqnarray*}

which expresses the mediation relationships in terms of expected outcomes.

FPCA plays a key role in transitioning the mediator definition from a single CpG site to a gene. FPCA not only aggregates site-level methylation signals within each gene but also reduces dimensionality while retaining biologically meaningful variation. A detailed explanation of this procedure follows.

### Extracting functional principal components

FPCA is a fundamental tool in functional data analysis, widely used for modeling data that exhibit spatial or temporal continuity. In this context, each gene can be regarded as an autonomous region whose constituent CpG sites display spatial dependence along the genomic coordinate. Treating each gene region as a distinct analytical domain, FPCA provides an effective and biologically justifiable approach to extract the principal functional features within each gene region. In this study, we compare two alternative methodologies for extracting informative components from each gene region. The dataset consists of *n* individuals whose genomes contain *N* genes. The CpG sites in the $i\mathrm{th}$ gene region are ordered as ${s}_{i1}\le{s}_{i2}\dots \le{s}_{i{m}_i}$. For individual *j=1,2,…, n*, let ${L}_{ij}=\left({L}_{ij}\left({s}_{i1}\right),\dots, {L}_{ij}\left({s}_{i{m}_i}\right)\right)$ represent the vector of methylation values in the $i\mathrm{th}$ gene region. Each element ${L}_{ij}\left({s}_{ij}\right)$ is defined as


$${L}_{ij}\left({s}_{ij}\right)={M}_{ij q},\kern0.5em {M}_{ij q}=\mathit{\ln}\left(\frac{\beta_{ij q}}{1-{\beta}_{ij q}}\right),q=1,\dots, {m}_i,$$

where ${\beta}_{ijq}$ denotes the DNAm *β*-value, representing the proportion of methylated signal intensity at site *q* of gene *i* for individual *j*. We define a continuous variant function ${G}_{ij}(s),s\in \left[1,{m}_i\right]$, to represent the methylation trajectory of the $i\mathrm{th}$ gene for individual *j*, estimated from the discrete vector ${L}_{ij}$. The collection of *n* trajectories on the $i\mathrm{th}$ gene is treated as *n* independent realizations of an integrable stochastic process $\left\{{G}_{ij}(s),s\in \left[1,{m}_i\right]\right\}$. We assume that this process has a mean function *μ (s)* and a covariance function $\mathrm{\nu} \left(s,l\right),s,l\in \left[1,{m}_i\right]$.

By Mercer's theorem, the covariance function admits the eigen-decomposition:


$$\mathrm{\nu} \left(s,l\right)={\sum}_{k=1}^{\infty }{\lambda}_k{\rho}_k(s){\rho}_k(l),$$

where ${\rho}_k$ and ${\lambda}_k$ are eigenfunctions and eigenvalues, respectively. According to the Karhunen–Loève theorem, the stochastic process can be represented as:


$${G}_{ij}(s)=\mu (s)+{\sum}_{k=1}^{\infty }{c}_{jk}{\rho}_k(s),$$

where the coefficients


$${c}_{ijk}={\int}_1^{m_i}\left({G}_{ij}(s)-\mu (s)\right){\rho}_k(s) ds$$

are the $k\mathrm{th}$ functional principal components $(FPC_s)$ for the $j\mathrm{th}$ trajectory. We retain the first $K_i$ eigenfunctions such that the cumulative proportion of variance explained satisfies


$$\frac{\sum_{k=1}^{K_i}{\lambda}_{ik}}{\sum_{k=1}^{\infty }{\lambda}_{ik}}\times 100\%\approx 85\%,$$

ensuring that the selected ${FPC}_s$ capture approximately 85% of the total variability.

To estimate each ${G}_{ij}(s)$, we employ typical basis functions, *B*-splines or Fourier series, to smooth the observed methylation profiles. The number of basis functions depends on the number of CpG sites within each gene and the basis's ability to reproduce the raw data. The $K_i$  *FPC* scores are then computed by numerical integration:


$${FPC}_{ijk}={\int}_1^{m_i}\left({G}_{ij}(s)-\mu_i (s)\right){\rho}_{ik}(s) ds,$$

where ${\rho}_{ik}(s)$ denotes the *k*th eigenfunction.

Between the two bases, *B*-spline fitting offers greater flexibility for capturing the heterogeneous and aperiodic structure of DNAm signals. In contrast, Fourier series are more suited for periodic phenomena and are less effective in modeling non-periodic patterns. Because DNAm signals lack inherent periodicity, we employ *B*-spline basis functions to achieve adaptive and smooth approximations that accommodate irregular site spacing and heterogeneous variability across gene regions. Each gene region is thus represented by ${K}_i$ uncorrelated ${FPC}_s$, which serve as gene-level summaries of the original methylation profiles. These *FPC* scores replace raw methylation vectors as new mediators in the subsequent mediation model.

We next extend the classical mediation model to incorporate the *FPC*-based mediators. If the outcome variable *Y* is continuous, the modified model (corresponding to Models (1)–(2)) is expressed as:


(3) \begin{eqnarray*} \left\{\begin{array}{@{}l}Y={\theta}_1+{c}_1X+{e}_1,\kern18.5em \\{}{FPC}_{ij}={\theta}_{2i}+{a}_{ih}+{e}_{2 ij},i=1,\dots, k;h=1,2;j=1,\dots, n,\\{}Y={\theta}_3+{c}_3X+{\sum}_{i=1}^k{b}_i{FPC}_i+{e}_3,\kern11.5em \end{array}\right. \end{eqnarray*}

And for a categorical outcome variable *Y*, taking the expectation yields:


(4) \begin{eqnarray*} \left\{\begin{array}{@{}l}E(Y)={\theta}_1+{c}_1X,\kern18.75em \\{}{FPC}_{ij}={\theta}_{2i}+{a}_{ih}+{e}_{2 ij},i=1,\dots, k;h=1,2;j=1,\dots, n,\\{}E(Y)={\theta}_3+{c}_3X+{\sum}_{i=1}^k{b}_i{FPC}_i.\kern11.5em \end{array}\right. \end{eqnarray*}

Here, ${\theta}_{2i}$ denotes the overall mean effect in the $i\mathrm{th}$ gene region, *h* represents different groups and ${a}_{ih}=\left({a}_{i,h=1},{a}_{i,h=2}\right)$ represents the group-specific mean effect vector in the *i*th gene.

To the best of our knowledge, while high-dimensional mediation analyses have been extensively studied, gene-based functional mediation modeling remains limited. By conceptualizing each gene region as an autonomous functional unit, FPCA effectively reduces intra-gene dimensionality and mitigates multicollinearity among CpG sites. Nonetheless, the number of gene-level mediators still exceeds the sample size, posing a high-dimensional estimation challenge. Because conventional regression approaches cannot reliably estimate or test the coefficients in the third equation of Models (3)–(4), we adopt a high-dimensional inference framework based on de-sparsified Lasso estimators [12]. The following sections provide detailed descriptions of the high-dimensional testing procedures and the methodological implementation of our proposed gHDMA model.

### Gene-set high-dimensional mediation analysis model

The steps and details of the gHDMA model are as follows:

#### Step 1: Extracting information

In the first step, FPCA was applied to extract the main information from each gene. The raw dataset was replaced by the corresponding *FPC* scores for every gene. Details of FPCA implementation are described elsewhere. After this step, we obtained a new dataset that contained only ${FPC}_s$ with no correlation within each gene region.

#### Step 2: Screening key genes

To identify genes with strong effects, we applied the Wilks' *\varLambda*-test to screen a certain number of genes based on the second equation in Model (3) or (4) [13]. This procedure follows the same principle as the sure independence screening (SIS) method proposed by Fan *et al.* [14]. Unlike SIS, which predefines the number of sites with the largest effects, our approach identifies genes showing the most significant effects in a data-driven manner. The number of retained genes depends on the response variable *Y*. When *Y* is continuous, the number of selected genes is $d=\left\lceil \frac{2n}{\mathit{\ln}(n)}\right\rceil\ (\left\lceil \cdot\right\rceil$ is the Ceiling function); when *Y* is binary, the number of reserved genes is set to one quarter of *d*. The screening ensures that no true genes are removed under theoretical assumptions. Screening is conducted based on the Wilks' *\varLambda*-test, where the primary purpose is to obtain the corresponding *P*-values. The *P*-values quantify the magnitude of mediation effects from *X* to ${FPC}_i,i=1,\cdots, k$.

The *P*-value can be computed as:


$${P}_{i,a}=\left\{1-{F}_{K_i,n-{K}_i-1}\left(\frac{\left(n-{K}_i-1\right)\left(1-{\varLambda}_i\right)}{K_i{\varLambda}_i}\right)\right\}\!,$$

where ${\varLambda}_i=\frac{\left|{\boldsymbol{W}}_{\boldsymbol{i}}\right|}{\left|{\boldsymbol{B}}_{\boldsymbol{i}}+{\boldsymbol{W}}_{\boldsymbol{i}}\right|}$ is the Wilks’*\varLambda* statistic, ${\boldsymbol{W}}_i={\sum}_{h=1}^2{n}_h\left({\hat{a}}_{ih}-{\hat{\theta}}_{2i}\right)\\{\left({\hat{a}}_{ih}-{\hat{\theta}}_{2i}\right)}^T$ and ${\boldsymbol{B}}_i={\sum}_{h=1}^2{\sum}_{j=1}^{n_h}\left({FPC}_{ij}-{\hat{a}}_{ih}\right){\left({FPC}_{ij}-{\hat{a}}_{ih}\right)}^T$. After screening, the number of genes is shrunk from *k* to *d*. We subsequently focused our analysis to these *d* genes to further evaluate their potential mediation effects with the method. Denote the remaining genes after the screening as ${FPC}_i,i=1,\cdots, d$.

#### Step 3: De-sparsified Lasso for computing *P*-values

After screening, we fit the following model:


(5) \begin{eqnarray*} E(Y)={\theta}_3+{c}_3X+{\sum}_{i=1}^d{b}_i{FPC}_{i{K}_i} \end{eqnarray*}

Since the dimension $D={\sum}_{i=1}^d{K}_i$ can still be relatively large after screening, ordinary least squares estimation is still infeasible. We therefore adopt a de-biased (or de-sparsified) estimator suitable for high-dimensional data [11]. Let ${\hat{b}}_{Lasso}$ denote the Lasso estimator. For a continuous response *Y*, the de-biased estimator, also called the de-sparsified estimator, is a bias-corrected estimator which is given as


(6) \begin{eqnarray*} {\hat{b}}_j=\frac{Z_j^TY}{Z_j^T{FPC}_j}-\sum_{l\ne j}\frac{Z_j^T{FPC}_l}{Z_j^T{FPC}_j}{\hat{b}}_{Lasso,l} \end{eqnarray*}

where ${\hat{b}}_j$ is the bias-corrected coefficient of the $j\mathrm{th}\ {FPC}_j$; ${\hat{b}}_{Lasso,l}$ is the coefficient of the $l\mathrm{th}\ {FPC}_l$; ${Z}_j$ is the regularized residuals obtained by ${Z}_j={FPC}_j-{FPC}_{-j}{\hat{\gamma}}_{Lasso}$, where ${\hat{\gamma}}_{Lasso}$ is the regression coefficients obtained based on a Lasso regression by regressing ${FPC}_j$ on all other *FPC*’s except the $j\mathrm{th}\ {FPC}_j$ denoted as ${FPC}_{-j}$. Van de Geer *et al.* [15] proved the asymptotic normality of the de-sparsified estimate, i.e.


$${u}_j=\frac{\sqrt{n}\left({\hat{b}}_j-{b}_j^0\right)}{\sigma_{\epsilon}\sqrt{\Omega_{jj}}}\overset{d}{\to }N\left(0,1\right)\kern0.75em as\kern0.75em p,n\to \infty$$

where ${b}_j^0$ represents the true regression coefficient; ${\sigma}_{\epsilon }$ can be calculated by using the scaled Lasso algorithm [16], and ${\Omega}_{jj}$ can be calculated by,


$${\Omega}_{jj}=\frac{n{Z}_j^T{Z}_j}{\left[{Z}_j^T{Z}_j\right]\left[{Z}_j^T{Z}_j\right]}.$$

Under the null that ${H}_0 \!\!:{b}_j^0=0$, *P*-values are computed from the asymptotic distribution.

For a binary response, van de Geer *et al.* also proved the asymptotic normality for the de-sparsified estimates. Let $W={\left(X, FPC\right)}^T,\boldsymbol{\beta} ={\left({c}_3,\boldsymbol{b}\right)}^T$, and ${L}_{\boldsymbol{\beta}}\left(y,W\right)=L\left(y,W\boldsymbol{\beta} \right)$ be a loss function and define ${\dot{L}}_{\beta }=\frac{\partial }{\partial \beta }{L}_{\beta }$ and ${\ddot{L}}_{\beta }=\frac{\partial^2}{\partial \beta \partial{\beta}^T}{L}_{\beta }$, and further define ${\varphi}_L:= {\sum}_{i=1}^nL\left({y}_i,{w_i}^T\boldsymbol{\beta} \right)/n$. The Lasso estimator for the mediation coefficients $\boldsymbol{\beta}$ is given as $\hat{\boldsymbol{\beta}}=\underset{\boldsymbol{\beta}}{argmin}\left({\varphi}_L+\lambda{\left\Vert \boldsymbol{\beta} \right\Vert}_1\right)$, where *λ* is a tuning parameter. Define $\hat{\Sigma}:= \varphi{\ddot{L}}_{\hat{\beta}}$ and construct $\hat{\Theta}={\hat{\Theta}}_{Lasso}$ by doing a nodewise Lasso with $\hat{\Sigma}$ as input. Then, the de-sparsified Lasso estimator is given as $\overset{\sim }{\boldsymbol{\beta}}:= \hat{\boldsymbol{\beta}}-\hat{\Theta}{\varphi}_{{\dot{L}}_{\hat{\beta}}}$. Van de Geer *et al.* [15] provided a detailed algorithm for computing the de-sparsified Lasso estimators in a generalized linear model framework. They also proved the asymptotic normality of the de-sparsified estimate, i.e.


$${u}_j=\frac{\sqrt{n}\left({\overset{\sim }{\beta}}_j-{\gamma}_j^0\right)}{{\hat{\sigma}}_j}\overset{\mathcal{d}}{\to }N\left(0,1\right)\kern0.75em as\kern0.75em p,n\to \infty$$

where ${\hat{\sigma}}_j^2={\left(\hat{\Theta}{\varphi}_{{\dot{L}}_{\hat{\beta}}{\dot{L}}_{\hat{\beta}}^T}{\hat{\Theta}}^T\right)}_{j,j}$. Similarly, we can get a *P*-value for each *FPC* based on the asymptotic normality property. Let the *P*-values for all the *D*  ${FPC}_s$ denoted as ${P}_g=\left({P}_{1,g},{P}_{2,g},\dots, {P}_{D,g}\right)$. ${P}_{j,g}$ can be calculated as ${P}_{j,g}=2\left\{1-\Phi \left(\frac{\sqrt{n}{\hat{b}}_j}{\sigma_{\epsilon}\sqrt{\Omega_{jj}}}\right)\right\}$ for a continuous *Y* or as ${P}_{j,g}=2\left\{1-\Phi \left(\frac{\sqrt{n}{\hat{b}}_j}{{\hat{\sigma}}_j}\right)\right\}$ for a discrete *Y*.

#### Step 4: *P*-value combination within each gene

To assess the overall significance of each gene region, we combine the *P*-values of all ${FPC}_s$ belonging to that region. Let ${P}_g=\left({P}_{1,g},{P}_{2,g},\dots, {P}_{D,g}\right)$ denote the vector of *P*-values for all *D*  ${FPC}_s$ of a given gene. These ${FPC}_s$ are partitioned into *d* contiguous regions, with the *j*th region containing ${K}_j$  ${FPC}_s$, such that $D={\sum}_{j=1}^d{K}_j$. We define ${\left\{P\right\}}_{K_j}$ as the vector of *P*-values for the ${FPC}_s$ within the *j*th region:


\begin{align*} {\left\{P\right\}}_{K_j}=\left\{\begin{array}{@{}l}\left(\underset{K_j}{\underbrace{P_{1,s},{P}_{2,s},\dots, {P}_{K_j,s}}}\right)\!, j=1,\kern12em \\{}\left(\underset{K_j}{\underbrace{P_{\sum_{j=1}^{j-1}{K}_j+1,s},{P}_{\sum_{j=1}^{j-1}{K}_j+2,s},\dots, {P}_{\sum_{j=1}^j{K}_j,s}}}\right)\!, j=2,3,\dots, d\ \end{array}\right. \end{align*}

where each vector ${\left\{P\right\}}_{K_j}$ has length ${K}_j$. Owing to the independence of ${FPC}_s$ within each region, when the number of ${FPC}_s$ in the *j*th region, ${K}_j\ge 2$, the Fisher's combination method is used to calculate the pooled *P*-value for that region [17]. Specifically, the statistic $-2\sum \mathit{\ln}\left({\left\{P\right\}}_{K_j}\right)$ follows a ${\chi}^2$ distribution with $2{K}_j$ degrees of freedom, based on which the *P*-value at the gene level can be obtained; if ${K}_j=1$, then the *P*-value of unique *FPC* within that region is directly taken as the *P*-value for that gene region.

#### Step 5: Joint significance test

To determine genes with significant mediation effects, we perform a joint test combining the *P*-values obtained from Step 2 (${P}_{i,a}$) and Step 4 $\left({P}_{j,g}\right)$. The joint test *P*-value is defined as:


$${P}_{t,M}=\mathit{\operatorname{Max}}\left({P}_{i,a},{P}_{j,g}\right), \; i,j,t\;correspond\;to\;the\;same\;gene.$$

A gene is considered to exhibit a significant mediation effect if ${P}_{t,M}<\alpha$, with significance level *α =0.05* [18].

## Simulation studies

### Simulation design

We conducted extensive simulations to evaluate the performance of the proposed gHDMA model. Datasets were generated according to Model (2), where each gene region represents a multi-site structure. The model was simplified as follows:


(7) \begin{eqnarray*} \left\{\begin{array}{@{}l}E(Y)={\theta}_1+{c}_1X\kern9.25em \\{}{FPC}_i={a}_2X+{e}_2,i=1,2,\dots, k.\kern3em \\{}E(Y)={c}_3X+{b}_3{\int}_0^1G(t) dt\kern4.5em \end{array}\right. \end{eqnarray*}

where the independent variable *X* was sampled from a binomial distribution, i.e. $X\sim B\left(n,0.5\right)$, and ${FPC}_i$ respresents the functional principal components summarizing gene region information. The functional trajectory *G(t)* was generated as $G(t)=\mu (t)+{\sum}_{i=1}^k{\phi}_i^j(t){FPC}_i+\varepsilon, i=1,\dots k;j=1,2$, where *μ (t)* is the mean function, ${\phi}_i^j(t)$ denotes eigenfunctions, and *ɛ* is random noise. Two types of basis functions were employed: (i) mutually orthogonal and (ii) mutually nonorthogonal. Specifically,


$${\phi}_i^1(t)=\left\{\sqrt{2}\sin \left(2\pi t\right),\sqrt{2}\cos \left(2\pi t\right),\sqrt{2}\sin \left(4\pi t\right),\sqrt{2}\cos \left(4\pi t\right)\right\}\!,$$

and


$${\phi}_i^2(t)=\Big\{1,\sqrt{3}\left(2t-1\right),\sqrt{5}\left(6{t}^2-6t+1\right),\sqrt{7}\left(20{t}^3-30{t}^2+12t-1\right)\!.$$

We considered four gene-level scenarios: (i) $\alpha \ne 0,\beta = 0$; (ii) $\alpha = 0,\beta \ne0$; (iii) $\alpha =0,\beta = 0$; and (iv) $\alpha \ne 0,\beta \ne 0$. The first three scenarios assess Type I error control, while the remaining one evaluates power. The parameter configurations were delineated for two cases, corresponding to binary and continuous outcome variables, respectively.

Case 1. Binary outcome variable:


$${\mathrm{Scenario}}_1\!:\left\{\begin{array}{@{}l}{a}_2={\left(0.20,0.30,0.40,0.50\right)}^T\ \\{}{b}_3={\left(0.00,0.00,0.00,0.00\right)}^T\ \end{array}\right.$$

$${\mathrm{Scenario}}_2\!:\left\{\begin{array}{@{}l}{a}_2={\left(0.00,\mathrm{0.00,0.00},0.00\right)}^T\ \\{}{b}_3={\left(0.25,0.25,0.25,0.25\right)}^T\ \end{array}\right.\!\!$$

$$\kern0.50em {\mathrm{Scenario}}_3\!:\left\{\begin{array}{@{}l}{a}_2={\left(0.00,0.00,0.00,0.00\right)}^T\ \\{}{b}_3={\left(0.00,0.00,0.00,0.00\right)}^T\ \end{array}\right.$$

\begin{align*} {\mathrm{Scenario}}_4 \!:\left\{\begin{array}{@{}l}{a}_2={\left(0.20,0.30,0.40,0.50\right)}^T\kern0.5em \\{}{b}_3={\left(0.25,0.25,0.25,0.25\right)}^T\kern0.5em \end{array}\right.\!\! \end{align*}

Case 2. Continuous outcome variable:


$${\mathrm{Scenario}}_1:\left\{\begin{array}{@{}l}{a}_2={\left(0.20,0.30,0.40,0.50\right)}^T\ \\{}{b}_3={\left(0.00,0.00,0.00,0.00\right)}^T\ \end{array}\right.$$

$${\mathrm{Scenario}}_2 \!:\left\{\begin{array}{@{}l}{a}_2={\left(0.00,\mathrm{0.00,0.00},0.00\right)}^T\ \\{}{b}_3={\left(0.11,0.11,0.11,0.11\right)}^T\ \end{array}\right.$$

$$\kern0.50em {\mathrm{Scenario}}_3 \!:\left\{\begin{array}{@{}l}{a}_2={\left(0.00,0.00,0.00,0.00\right)}^T\ \\{}{b}_3={\left(0.00,0.00,0.00,0.00\right)}^T\ \end{array}\right.$$

$${\mathrm{Scenario}}_4 \!:\left\{\begin{array}{@{}l}{a}_2={\left(0.20,0.30,0.40,0.50\right)}^T\kern0.5em \\{}{b}_3={\left(0.11,0.11,0.11,0.11\right)}^T\kern0.5em \end{array}\right. \!\!$$

We further examined different sample sizes (*n=200,400*) and numbers of sites per gene region (*m=50,100,500*). In addition, a more representative setting, partial mediation with direct effect ${c}_3=0.2,$was analyzed. Each gene region was simulated as:


$$G(t)={\sum}_{i=1}^4{\phi}_i^j(t){FPC}_i+\varepsilon, j=1,2,$$

where $\varepsilon \sim MVN(0,I)$. For binary outcomes,


$${Y}_i\sim Bernoulli\left({P}_i\right),\kern0.5em {P}_i=\frac{\mathit{\exp}\left({\eta}_i\right)}{1+\mathit{\exp}\left({\eta}_i\right)},\kern0.5em {\eta}_i={c}_3X+{b}_3{\int}_0^1G(t) dt,$$

And for continuous outcomes,


$${Y}_i={c}_3X+{b}_3{\int}_0^1G(t) dt+{\varepsilon}_3,{\varepsilon}_3\sim N\left(0,1\right)$$

We evaluated the performance of the gHDMA model in terms of false positive control and power and reported the results in Table 1 to Table 4. Each configuration was replicated 1000 times. For each dataset, *P*-values of ${FPC}_s$ were obtained via multiple linear regression and combined using Fisher's *P*-value combination method to compute the gene-level *P*-value.

**Table 1:** List of Type I error rates under the three scenarios when the outcome is binary.

Scenario	*n*	*m*	Basis function	Type I error rate
$\alpha \ne 0,\beta =0$	200	50	Orthogonal	0.058
			Nonorthogonal	0.058
		100	Orthogonal	0.060
			Nonorthogonal	0.054
		500	Orthogonal	0.059
			Nonorthogonal	0.056
	400	50	Orthogonal	0.055
			Nonorthogonal	0.054
		100	Orthogonal	0.049
			Nonorthogonal	0.048
		500	Orthogonal	0.051
			Nonorthogonal	0.056
$\alpha =0,\beta \ne 0$	200	50	Orthogonal	0.039
			Nonorthogonal	0.053
		100	Orthogonal	0.044
			Nonorthogonal	0.043
		500	Orthogonal	0.039
			Nonorthogonal	0.046
	400	50	Orthogonal	0.045
			Nonorthogonal	0.048
		100	Orthogonal	0.046
			Nonorthogonal	0.048
		500	Orthogonal	0.048
			Nonorthogonal	0.049
$\alpha =0,\beta =0$	200	50	Orthogonal	0.004
			Nonorthogonal	0.001
		100	Orthogonal	0.001
			Nonorthogonal	0.001
		500	Orthogonal	0.002
			Nonorthogonal	0.001
	400	50	Orthogonal	0.003
			Nonorthogonal	0.006
		100	Orthogonal	0.002
			Nonorthogonal	0.003
		500	Orthogonal	0.002
			Nonorthogonal	0.002

**Table 2:** List of Type I error rates under the three scenarios when the outcome is continuous.

Scenario	*n*	*m*	Basis function	Type I error rate
$\alpha \ne 0,\beta =0$	200	50	Orthogonal	0.050
			Nonorthogonal	0.045
		100	Orthogonal	0.052
			Nonorthogonal	0.044
		500	Orthogonal	0.049
			Nonorthogonal	0.045
	400	50	Orthogonal	0.042
			Nonorthogonal	0.043
		100	Orthogonal	0.047
			Nonorthogonal	0.041
		500	Orthogonal	0.046
			Nonorthogonal	0.044
$\alpha =0,\beta \ne 0$	200	50	Orthogonal	0.029
			Nonorthogonal	0.034
		100	Orthogonal	0.032
			Nonorthogonal	0.033
		500	Orthogonal	0.032
			Nonorthogonal	0.033
	400	50	Orthogonal	0.047
			Nonorthogonal	0.047
		100	Orthogonal	0.046
			Nonorthogonal	0.050
		500	Orthogonal	0.047
			Nonorthogonal	0.047
$\alpha =0,\beta =0$	200	50	Orthogonal	0.002
			Nonorthogonal	0.003
		100	Orthogonal	0.003
			Nonorthogonal	0.003
		500	Orthogonal	0.002
			Nonorthogonal	0.002
	400	50	Orthogonal	0.002
			Nonorthogonal	0.002
		100	Orthogonal	0.002
			Nonorthogonal	0.002
		500	Orthogonal	0.001
			Nonorthogonal	0.002

**Table 3:** List of power under the scenario when the outcome is binary.

Scenario	*n*	*m*	Basis function	Power
$\alpha \ne 0,\beta \ne 0$	200	50	Orthogonal	0.775
			Nonorthogonal	0.748
		100	Orthogonal	0.770
			Nonorthogonal	0.766
		500	Orthogonal	0.778
			Nonorthogonal	0.774
	400	50	Orthogonal	0.965
			Nonorthogonal	0.966
		100	Orthogonal	0.969
			Nonorthogonal	0.966
		500	Orthogonal	0.971
			Nonorthogonal	0.966

**Table 4:** List of power under the scenario when the outcome is continuous.

Scenario	*n*	*m*	Basis function	Power
$\alpha \ne 0,\beta \ne 0$	200	50	Orthogonal	0.670
			Nonorthogonal	0.674
		100	Orthogonal	0.689
			Nonorthogonal	0.669
		500	Orthogonal	0.681
			Nonorthogonal	0.677
	400	50	Orthogonal	0.952
			Nonorthogonal	0.966
		100	Orthogonal	0.953
			Nonorthogonal	0.963
		500	Orthogonal	0.960
			Nonorthogonal	0.963

### Simulation results

#### Type I error control

There are several patterns observed from Tables 1 and 2. (i) The choice of basis function has a minimal impact on the analysis results. In almost all cases, the use of orthogonal or non-orthogonal basis functions exerts little influence on Type I error control. For instance, in Scenario 1 ($\alpha \ne 0,\beta =0$) with a continuous outcome, *n=200* and *m=50*, the empirical Type I error rates are 0.050 for the orthogonal function and 0.045 for the nonorthogonal function, corresponding to a negligible difference of only 0.005. (ii) The Type I error is effectively controlled at the nominal significance level (*α =0.05*) in most cases. For Scenarios 1 and 2 ($\alpha \ne 0$ or $\beta \ne 0$), regardless of the sample size *n*, number of sites *m*, or the type of basis function, the empirical Type I error rates for both binary and continuous outcomes consistently converge to 0.05. As an illustration, in Table 2 (continuous outcome), the empirical Type I error rates for these two scenarios mostly range from 0.029 to 0.052, which demonstrates satisfactory Type I error control. (iii) The test exhibits conservatism under the global null hypothesis (Scenario 3, $\alpha =0,\beta =0$) for both binary and continuous outcomes. This phenomenon can be attributed to the random independence between *α =0* and *β =0* in this scenario: the nominal Type I error rate is set at 0.05 for both individual tests of *α* and *β*, while the test in Model (7) yields a Type I error only when both $\alpha \ne 0$ and $\beta \ne 0$ simultaneously. Under the assumption of independence between the tests for *α* and *β*, the theoretically expected Type I error rate of Model (7) should be approximately 0.0025 (i.e. 0.05^2^), and all the Type I error rates align with this theoretical value.

Figure 2 assesses the Type I error control of the gHDMA model for a continuous outcome, examining the interplay between sample size (*n*), the number of sites (*m*), and the choice of basis function design (Orthogonal vs. Nonorthogonal). Overall, the empirical Type I error rates are well controlled around the nominal 0.05 level across all conditions. The control becomes more precise and stable as the sample size increases from *n=200* to *n=400*, with error rates converging closely to 0.05. Furthermore, the Nonorthogonal design yields marginally more conservative (slightly lower) error rates compared to the Orthogonal design when the number of sites is small (e.g. *m=50*), but this subtle difference diminishes as *m* increases to 100 and 500. Figure 3 further evaluates Type I error control by decomposing it into three specific null scenarios (Scenarios 1, 2, and 3). The results demonstrate that the model reliably controls the Type I error near the 0.05 target in Scenarios 1 and 2, where partial null hypotheses are tested. In contrast, under the complete null hypothesis of Scenario 3, the model produces slightly conservative (lower than 0.05) error rates. This pattern is consistent across different sample sizes and numbers of sites.

**Figure 2:**
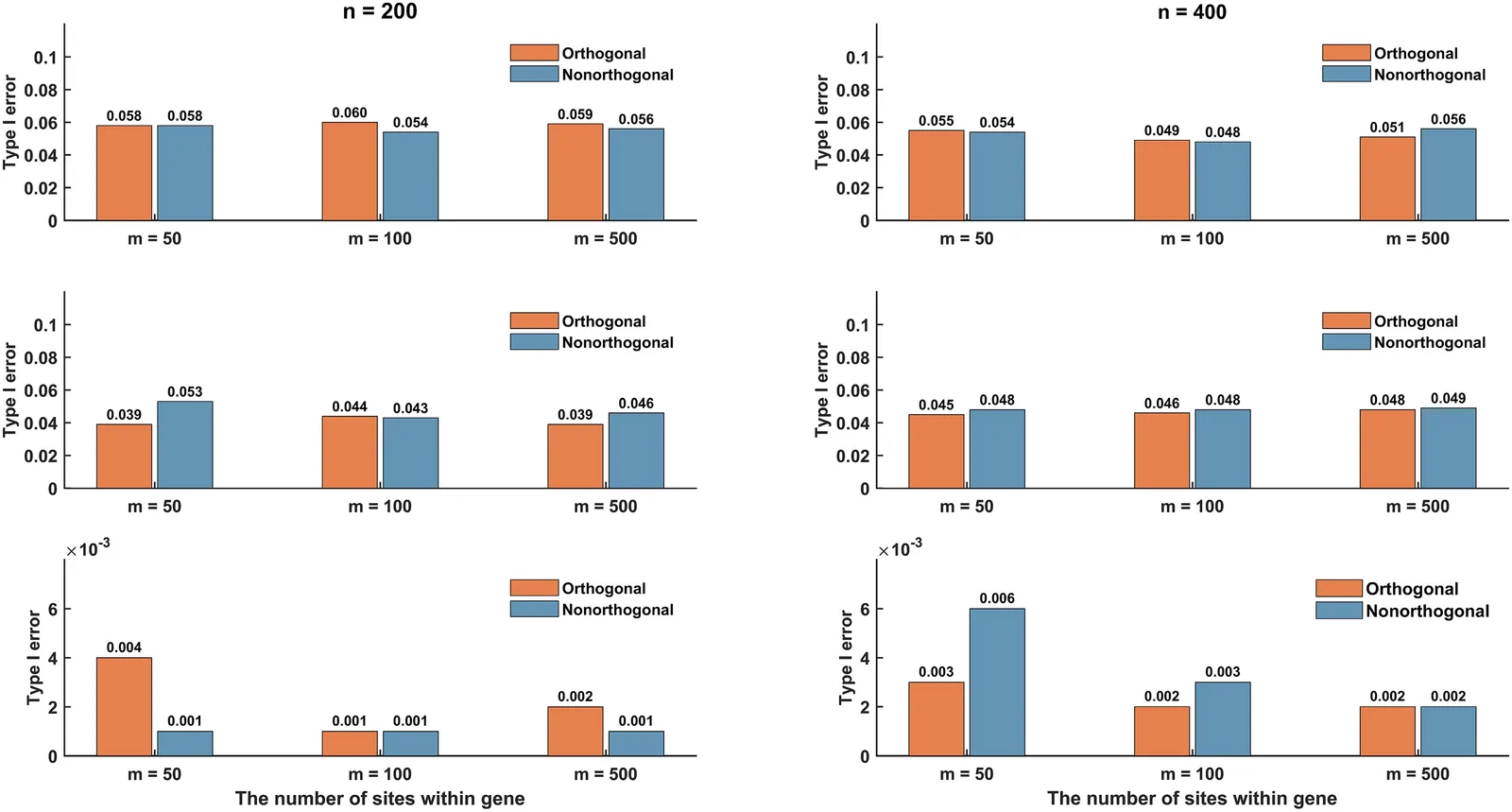
Type I error rates of the gHDMA model for binary outcomes under null-mediation-effect simulation scenarios, across varying sample sizes, numbers of sites, types of basis functions, and scenarios. The top two panels show the Type I error rates for the case $\alpha \ne 0,\beta =0$; the middle two panels display the results for $\alpha =0,\beta \ne 0$; and the bottom two panels present the findings for $\alpha =0,\beta =0$.

**Figure 3:**
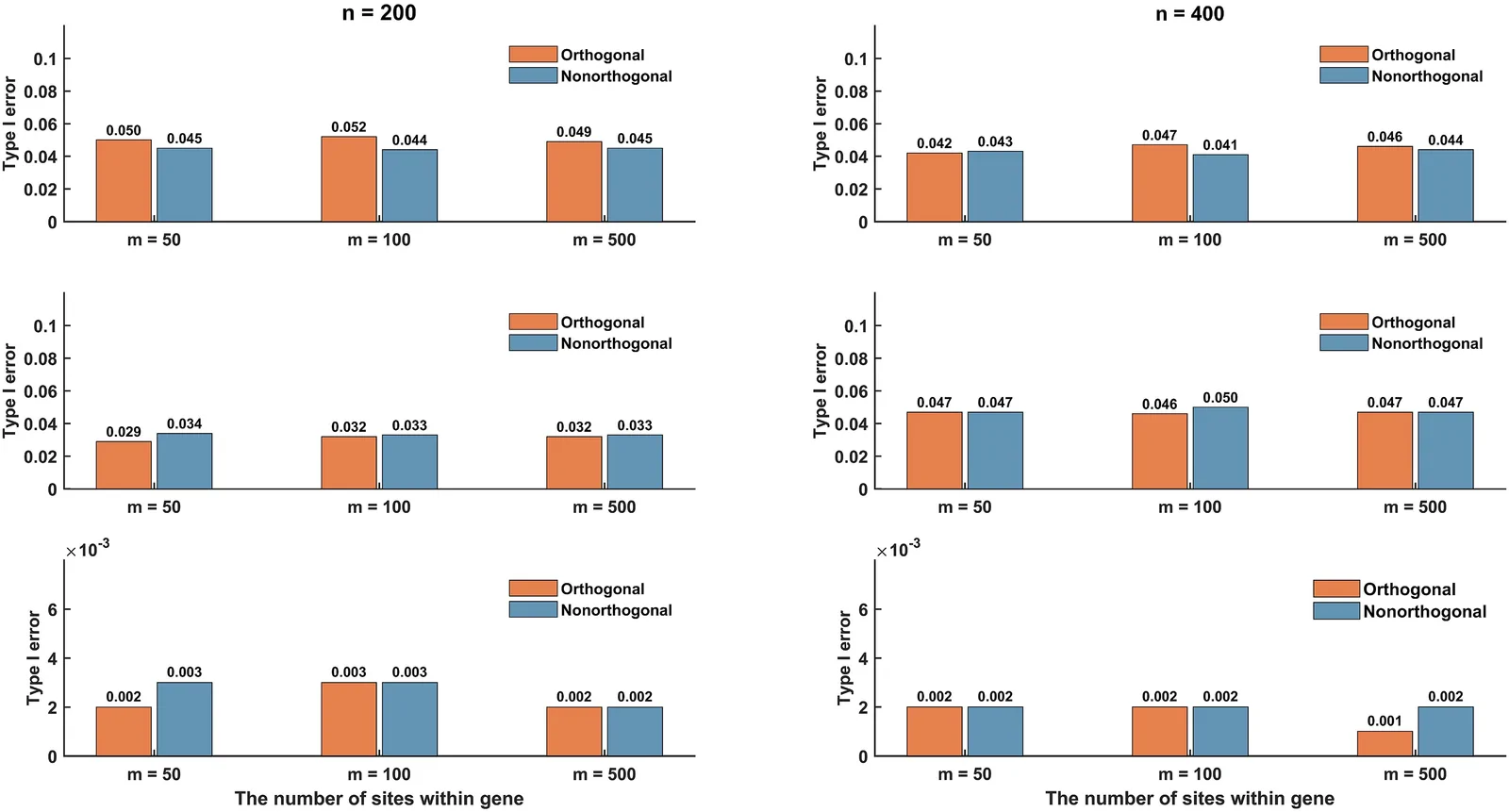
Type I error rates of the gHDMA model for continuous outcomes under null-mediation-effect simulation scenarios, across varying sample sizes, numbers of sites, types of basis functions, and scenarios. The top two panels show the Type I error rates for the case $\alpha \ne 0,\beta =0$; the middle two panels display the results for $\alpha =0,\beta \ne 0$; and the bottom two panels present the findings for $\alpha =0,\beta =0$.

#### Statistical power

Several clear patterns can be observed from Tables 3 and 4. (i) The choice of basis function has a minimal impact on statistical power. Across all combinations of *n* and *m*, the power of gHDMA model for orthogonal and nonorthogonal basis functions are remarkably close. The largest observed difference is 0.027 (0.775 vs. 0.748 for binary outcome, *n=200*, *m=50*, in Table 3). In most settings, especially with larger sample sizes (*n=400*), the difference is negligible, often less than 0.01. (ii) Sample size (*n*) is the dominant factor influencing power. Increasing the *n* from 200 to 400 leads to a substantial and consistent increase in power for all configurations. For instance, with a binary outcome and *m=50* (Table 3), the power of gHDMA model jumps from approximately 0.76–0.78 to over 0.96. A similar dramatic increase is observed for continuous outcomes (Table 4), where power rises from around 0.67–0.69 to over 0.95. (iii) Increasing the *m* has a very limited effect on the power of gHDMA model, particularly at larger *n*. When *n=200*, the power of gHDMA model shows slight fluctuations as *m* increases from 50 to 500, but without a clear directional trend. When *n=400*, power stabilizes at a high level, showing only minimal incremental changes (e.g. from 0.965 to 0.971 for orthogonal functions in Table 3).

Figure 4 illustrates the statistical power of the gHDMA model for a binary outcome. The results demonstrate that power is strongly dependent on *n*, showing a major improvement from *n=200* to *n=400*. In contrast, increasing the number of sites within the gene (*m*) has a negligible effect on power, as values quickly plateau. Figure 5 displays the corresponding power for a continuous outcome. It reveals an identical trend: sample size is the dominant factor for increasing power, while the number of sites (*m*) and the choice of basis function (orthogonal vs. nonorthogonal) have minimal impact. A direct comparison of the two figures shows that the model achieves slightly higher power for binary outcomes than for continuous outcomes at the smaller sample size (*n=200*), but both achieve similarly high power (*>0.95*) when *n=400*.

**Figure 4:**
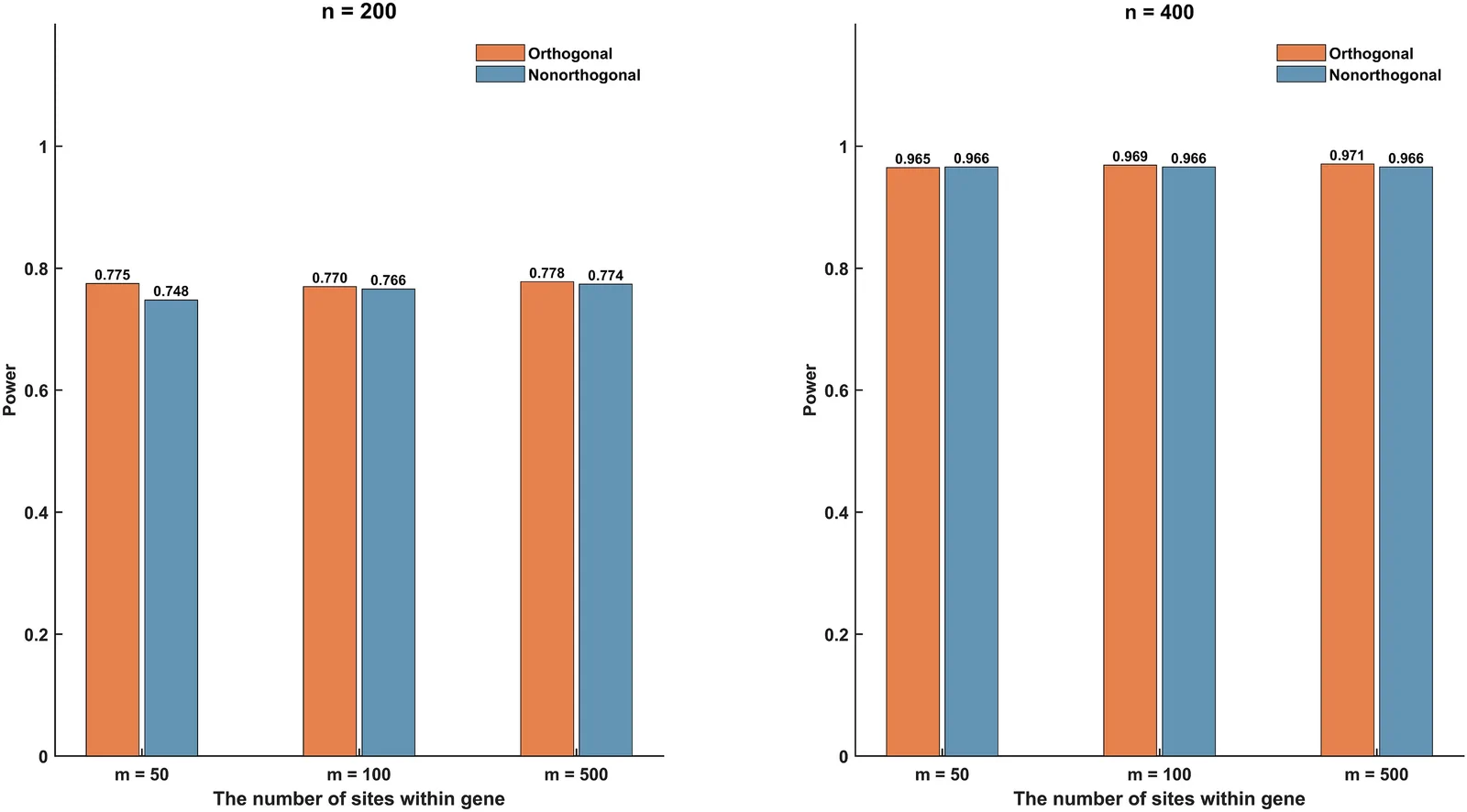
Power of the gHDMA model for binary outcomes under non-null-mediation-effect simulation scenario, across varying sample sizes, numbers of sites, and types of basis functions.

**Figure 5:**
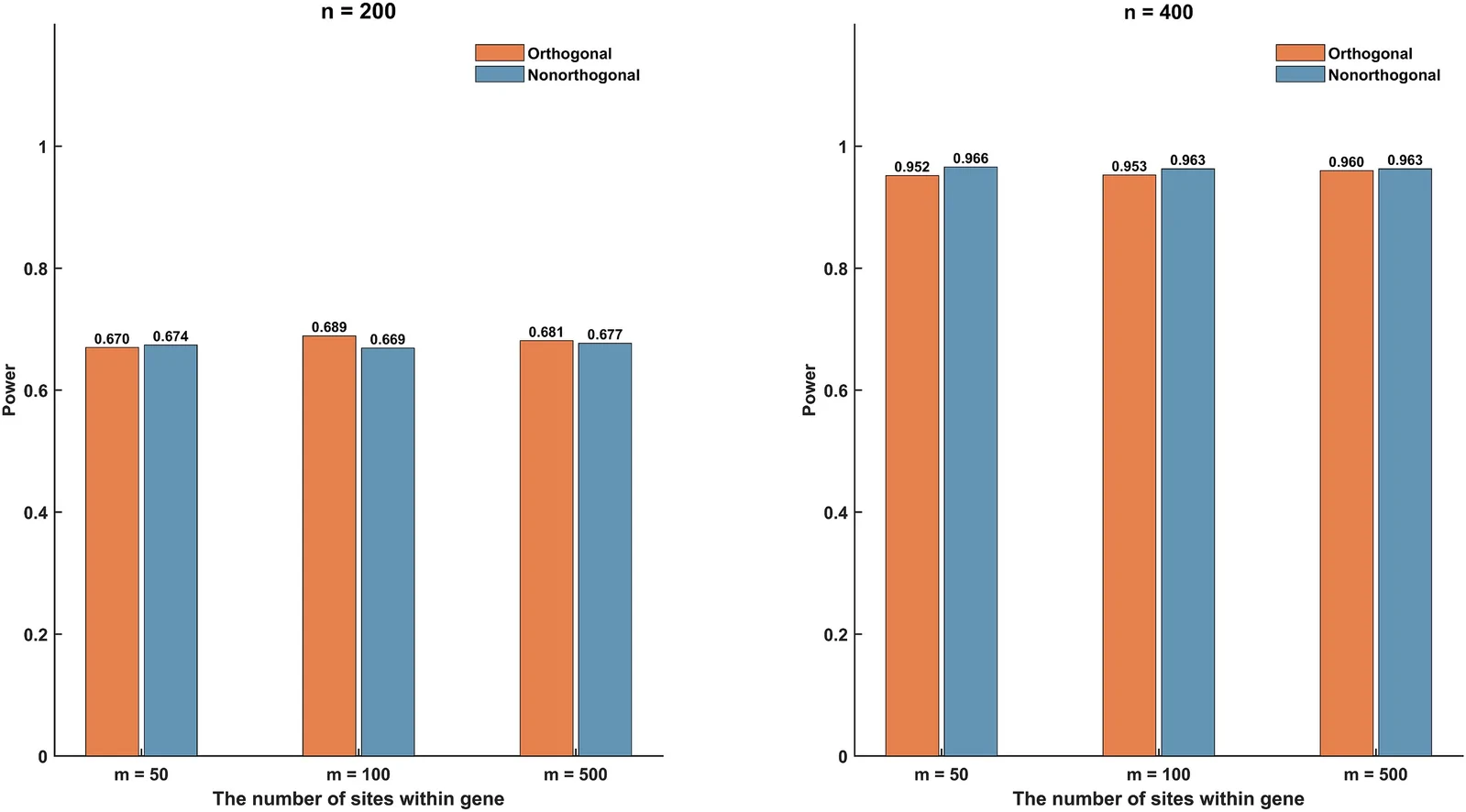
Power of the gHDMA model for continuous outcomes under non-null-mediation-effect simulation scenario, across varying sample sizes, numbers of sites, and types of basis functions.

Overall, across all simulation settings, the proposed gHDMA model performs well. Notably, both the power and Type I error rate tend to plateau once the number of sites reaches a sufficiently large value. The conservative behavior observed is expected due to the use of a joint significance test. In Scenarios 1 and 2, one of the effects is set to zero, and the Type I error rates of the gHDMA model remain close to the 0.05 level, with false positives appearing randomly across the 1000 simulation replicates. In Scenario 3, where both effects are set to zero, false positives arise purely by chance during the testing steps. Because of this randomness, the results from the two-step testing procedure are not perfectly synchronized. Consequently, the overall Type I error rate of the gHDMA model is reasonably controlled at approximately 0.0025.

## Real data analysis

We apply the gHDMA model to a real dataset to identify methylation genes that may serve as underlying mediators. DNAm plays a key role in numerous diseases, highlighting the importance of improving our understanding of pathogenic mechanisms [19]. In this analysis, we evaluate the effect of childhood maltreatment on adult depression, mediated by gene-level DNAm. DNAm may represent a biological mechanism through which early traumatic experiences, such as childhood maltreatment, become embedded and influence later-life outcomes [20].

The data were obtained from the Grady Trauma Project (GTP), which recruited African American participants from inner-city Atlanta. The study was approved by the Institutional Review Board of the Emory University School of Medicine and Grady Memorial Hospital [21]. A growing body of research suggests that DNAm serves as a key intermediary linking environmental exposures to the development of depressive disorders. Early-life adversities, particularly childhood maltreatment, have been shown to induce substantial disruptions in biological developmental processes, leading to a wide range of downstream consequences. Epigenetic modifications, with DNAm as a central component, are increasingly recognized as fundamental mechanisms underlying these deviations from typical development [22].

In this study, we apply the proposed methodology to characterize the relationship between childhood maltreatment and adult depression, with particular attention to the mediating role of DNAm at the gene level. The dataset includes baseline demographic information, estimated cell-type composition, and DNAm measurements. Depression status is determined using the Beck Depression Inventory (BDI), a validated psychometric tool used to classify individuals into case and control groups. Participants with current depressive symptoms are defined as cases if their BDI score is ≥14, whereas controls are defined as individuals with a BDI score <14 despite experiencing trauma exposure [20]. We remove observations with missing values and exclude individuals receiving depression treatment, as such treatment may alter DNAm patterns and complicate mediation analyses. After these exclusions, 138 cases and 145 controls remain for analysis. The overall data analysis steps applied to this cohort is depicted in Fig. 6.

**Figure 6:**
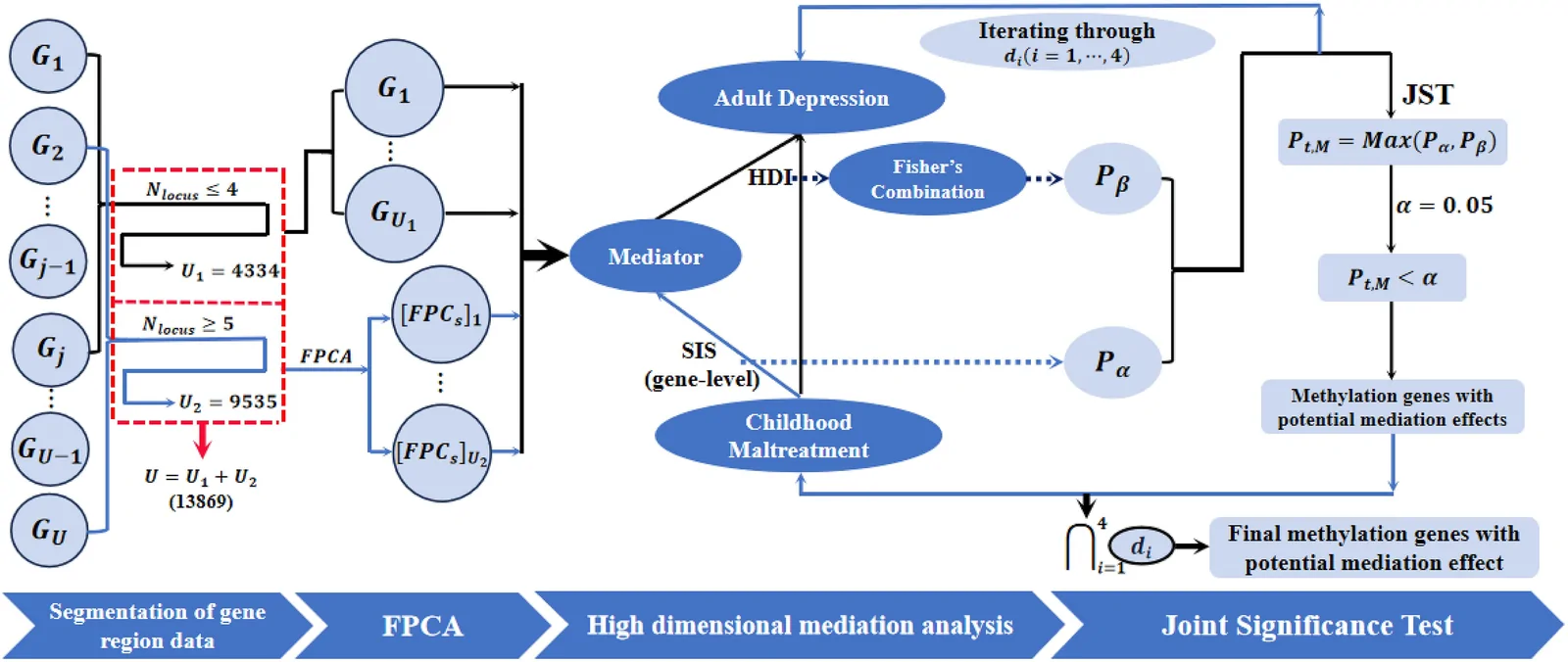
Analytical workflow for mediation genes identification using the gHDMA model in the GTP datasets.

Table 5 summarizes the demographic characteristics of the study population. The age ranges for cases and controls are (18, 70) and (18, 74), respectively. No statistically significant differences are observed across age, sex, or Body Mass Index (BMI); however, the prevalence of moderate-to-extreme childhood sexual/physical abuse (Childhood Maltreatment) is significantly higher in cases than in controls. Since none of the examined clinical variables show significant differences, we do not include additional covariates in the gHDMA model.

**Table 5:** Partial list of covariates and their associations with depression case/control status.

Covariates	Case (*N* = 138)	Control (*N* = 145)	Total (*N* = 283)	*P*-value
**Age**				
Mean(SD)	40.19(12.74)	41.45(13.47)	40.83(13.11)	.420
**Sex** $n\left(\%\right)$				
Male	97(70.29)	102(70.34)	199(70.32)	1.000
Female	41(29.71)	43(29.66)	84(29.68)	
**BMI** (kg/m^2^)				
Mean(SD)	32.42(9.27)	32.22(7.57)	32.32(8.43)	.846
**Childhood sexual/physical abuse** **moderate to extreme** $n\left(\%\right)$				
No	67(48.55)	99(68.28)	166(58.66)	.0012
Yes	71(51.45)	46(31.72)	117(37.39)	

**Table 6:** List of significant genes identified by gHDMA model under distinct scenarios.

The number of genes retained	The number of gene identifications	Chromosome	Gene name	*P*-value	Gene biological function
${d}_1=26$	3	7	***WNT2***	.0089	Regulates hippocampal neurogenesis and plasticity via WNT/β-catenin signaling; its downregulation under stress increases depression susceptibility [23, 28].
		20	***RAE1***	.0438	Controls mRNA nucleocytoplasmic transport to sustain neuronal plasticity [24, 25].
		21	***RUNX1***	.0135	Modulates neurogenesis and immune homeostasis via BDNF regulation and neuroinflammation, mediating depression through gene–environment interactions [26, 27].
${d}_2=51$	5	7	***WNT2***	.0092	Same as above
		20	***RAE1***	.0330	Same as above
		21	***RUNX1***	.0285	Same as above
		1	*VPS13D*	.0414	A key lipid transfer protein regulating mitochondrial homeostasis and autophagy; its dysfunction induces neuronal autophagy arrest and axonal damage, causing motor, cognitive and neuropsychiatric disorders [29, 30].
		19	*EIF3K*	.0167	Core subunit of the EIF3 complex that represses selective mRNA translation, autophagy and apoptosis. Its dysfunction disrupts neurodevelopment, leading to microcephaly and developmental disorders [31, 32].
${d}_3=101$	10	7	***WNT2***	.0096	Same as above
		20	***RAE1***	.0209	Same as above
		21	***RUNX1***	.0339	Same as above
		1	*VPS13D*	.0303	Same as above
		19	*EIF3K*	.0096	Same as above
		4	*RAB33B*	.0300	A core member of the Rab small G-protein family localized at the Golgi apparatus. It initiates autophagy via direct binding to Atg16L1 to promote autophagosome formation, and its dysfunction causes skeletal dysplasia and neural homeostasis imbalance [33, 34].
		19	*RPS5*	.0307	A core protein of the 40S small ribosomal subunit that maintains accurate mRNA-tRNA pairing to ensure translational fidelity. Its mutation or epigenetic alteration impairs translation accuracy, reduces cellular stress tolerance and induces abnormal neurodevelopment [35].
		21	*SPATC1L*	.0380	It encodes a centrosome-associated protein sensitive to environmental stress. Its epigenetic dysregulation contributes to PTSD and major depression [36].
		21	*BRWD1*	.0146	Acts as an epigenetic reader to regulate chromatin remodeling and cell differentiation [37].
		22	*SLC5A1*	.0047	A sodium–glucose cotransporter mediating glucose absorption and endothelial protection under stress [38].
${d}_4=201$	16	7	***WNT2***	.0116	Same as above
		20	***RAE1***	.0354	Same as above
		21	***RUNX1***	.0190	Same as above
		19	*EIF3K*	.0102	Same as above
		4	*RAB33B*	.0190	Same as above
		19	*RPS5*	.0238	Same as above
		21	*SPATC1L*	.0249	Same as above
		21	*BRWD1*	.0206	Same as above
		22	*SLC5A1*	.0033	Same as above
		1	*ACBD6*	.0417	A myristoyl-CoA binding protein that regulates protein N-terminal myristoylation. Its variants cause neurodevelopmental syndromes [39].
		3	*OXSM*	.0251	A rate-limiting enzyme for mitochondrial fatty acid synthesis that modulates OXPHOS and nutrient sensing [40].
		6	*GLP1R*	.0379	GLP-1 receptor exerting anti-inflammatory and protective effects in the nervous and immune systems [41].
		6	*GRM7*	.0156	A presynaptic metabotropic glutamate receptor that controls synaptic excitability via negative feedback regulation [42].
		6	*RIMKLB*	.0258	NAAG synthetase II that catalyzes the synthesis of the neuropeptide NAAG in the brain [43].
		11	*RBM4*	.0151	A selective RNA-binding protein that modulates neural differentiation by regulating RNA splicing [44].
		19	*CYP4F11*	.0154	A cytochrome P450 ω-hydroxylase involved in anti-inflammatory lipid metabolism [45].

Raw methylation beta values from the HumanMethylation450k BeadChip (Illumina) were obtained using the Illumina BeadStudio software. We excluded samples with probe detection call rates *<90%* or with an average intensity value *<50%* of the study-wide mean or *<2000* arbitrary units (AU). Beta values were logit-transformed to *M*-values, and 335 669 CpG sites were retained for downstream processing. After mapping CpG sites to annotated genes, we obtained a total of 13 869 genes. Among them, 9535 genes contained at least five CpG sites, and the remaining 4334 genes had fewer than five CpG sites. Functional principal components (${FPC}_s$) accounting for at least 90% of total variation were extracted for each gene region containing ≥5 CpG sites and were used in place of raw CpG sets in the gHDMA model. By contrast, gene regions with fewer than five CpG sites were directly incorporated into the model using their original methylation values. Readers may refer to https://gradytraumaproject.com/ for additional information on the dataset. The raw data are available for download at: https://www.ncbi.nlm.nih.gov/geo/query/acc.cgi?acc=GSE72680.

We applied the proposed gene-level mediation analysis to identify genes mediating the effect of childhood maltreatment on adult depression. The mediation model was fitted while adjusting for cell-type composition whenever ${FPC}_s$ were included:


$$\left\{\begin{array}{@{}l} logit(P)={\theta}_1+{c}_{Maltreament} Maltreatment\kern12.5em \\{}{FPC}_j={\theta}_j^{\prime }+{a}_j Maltreatment+{\lambda}_1^T Cell+{\varepsilon}_j,j=1,\dots, k.\kern6.5em \\{} logit(P)={\theta}_2+{c}_{Maltreatment}^{\prime } Maltreatment+{\sum}_{j=1}^k{b}_j{FPC}_j+{\lambda}_2^T Cell\end{array}\right.$$

Using the first regression equation, we estimated the total effect of childhood maltreatment on depression as ${\hat{c}}_{Maltreatment}=0.8245$(95% CI: [0.344, 1.313]). During the SIS screening step, we retained four different quantities of mediators including ${d}_i\in \left\{\left\lceil \frac{n}{2\mathit{\ln}(n)}\right\rceil \!, \left\lceil \frac{n}{\mathit{\ln}(n)}\right\rceil \!, \left\lceil \frac{2n}{\mathit{\ln}(n)}\right\rceil \!,\right. \left. \left\lceil \frac{4n}{\mathit{\ln}(n)}\right\rceil \right\} \!,i=1,2,3,4$ (where $\left\lceil \cdot\right\rceil$denotes the ceiling function and *n* denotes the sample size) to evaluate the robustness of the final results, given our relatively small sample size. We performed SIS filtering and independent mediation analysis under each of the four scenarios. Genes identified across all four settings were retained by taking their intersection, yielding the final set of candidate methylation genes with potential mediation effects. High-dimensional inference was applied to the final regression model. All results are summarized in Table 6. No genes remained significant after controlling the false discovery rate at the 0.05 level; therefore, we report the three robust candidate genes identified from the intersection analysis (as shown in Fig. 7) that reached suggestive significance.

**Figure 7:**
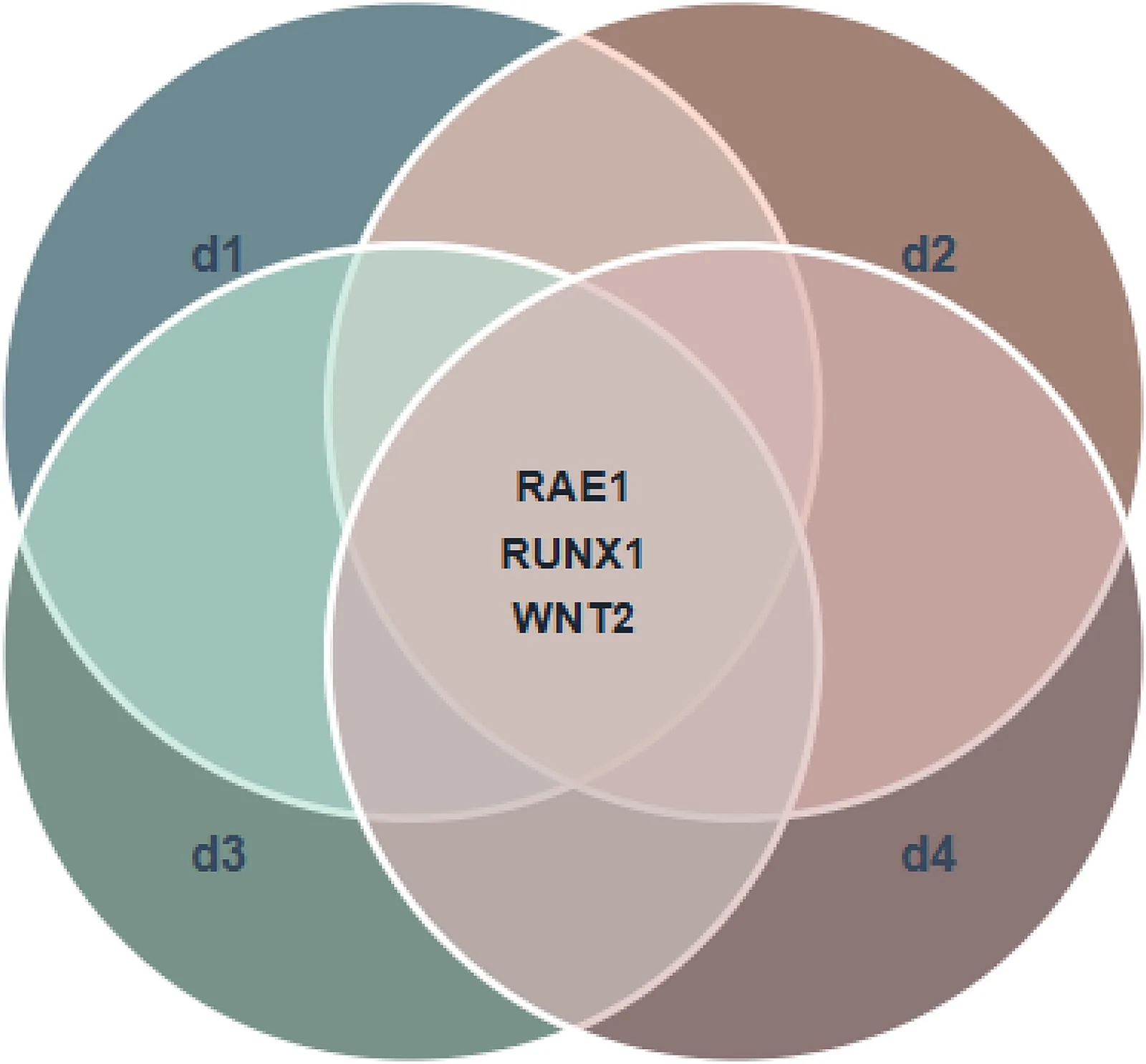
Three methylation genes obtained by taking the intersection of results from four SIS screening scenarios for childhood-maltreatment-related adult depression mediation analysis.

As shown in Table 6 and Fig. 7, three genes, *WNT2, RAE1*, and *RUNX1*, were identified as potential mediators of the effect of childhood maltreatment on adult depression, located on chromosomes 7, 20 and 21, respectively. Several lines of biological evidence support their potential roles. Specifically, *WNT2* activates *WNT*/*β*-catenin signaling to support hippocampal neurogenesis, and its reduction in the ventral hippocampus drives stress-induced depression-like behaviors [23]. *RAE1* is a well-established regulator of mRNA nuclear export, a process dynamically modulated by cells to cope with environmental stress. We posit that this Rae1-dependent mechanism is equally critical for preserving neuronal synaptic plasticity under chronic CNS stress. Dysregulation of this export pathway could impair the synthesis of key synaptic proteins, thereby driving the pathogenesis of adult depression [24, 25]. *RUNX1* contributes to the pathogenesis of depression through both central neural plasticity and peripheral immune regulation. In adult hippocampal excitatory neurons, *RUNX1* forms a transcriptional complex with CBF*β* to maintain protein stability. Loss of this complex impairs BDNF expression, which subsequently disrupts hippocampal neurogenesis—particularly in females—thereby reducing stress resilience and precipitating anxiety- and depression-like behaviors. Furthermore, as a key regulator of myeloid differentiation, *RUNX1* has been significantly associated with depression risk in large-scale genetic studies. Its genetic variants exert gene–environment interactions with childhood maltreatment to drive macrophage activation and systemic immune remodeling. Such peripheral-central immune crosstalk induces low-grade neuroinflammation, collectively fueling the pathological progression of depression [26, 27].

Figure 8 presents the line plots of methylation signals for the three gene regions. The gray lines show the *M*-values for individual samples across each gene region, while the blue lines represent the average methylation trajectories obtained through *B*-spline functionalization. As illustrated in the figure, the mean methylation patterns for *WNT2* exhibit nonlinear trends, whereas those for *RAE1* and *RUNX1* appear approximately linear.

**Figure 8:**
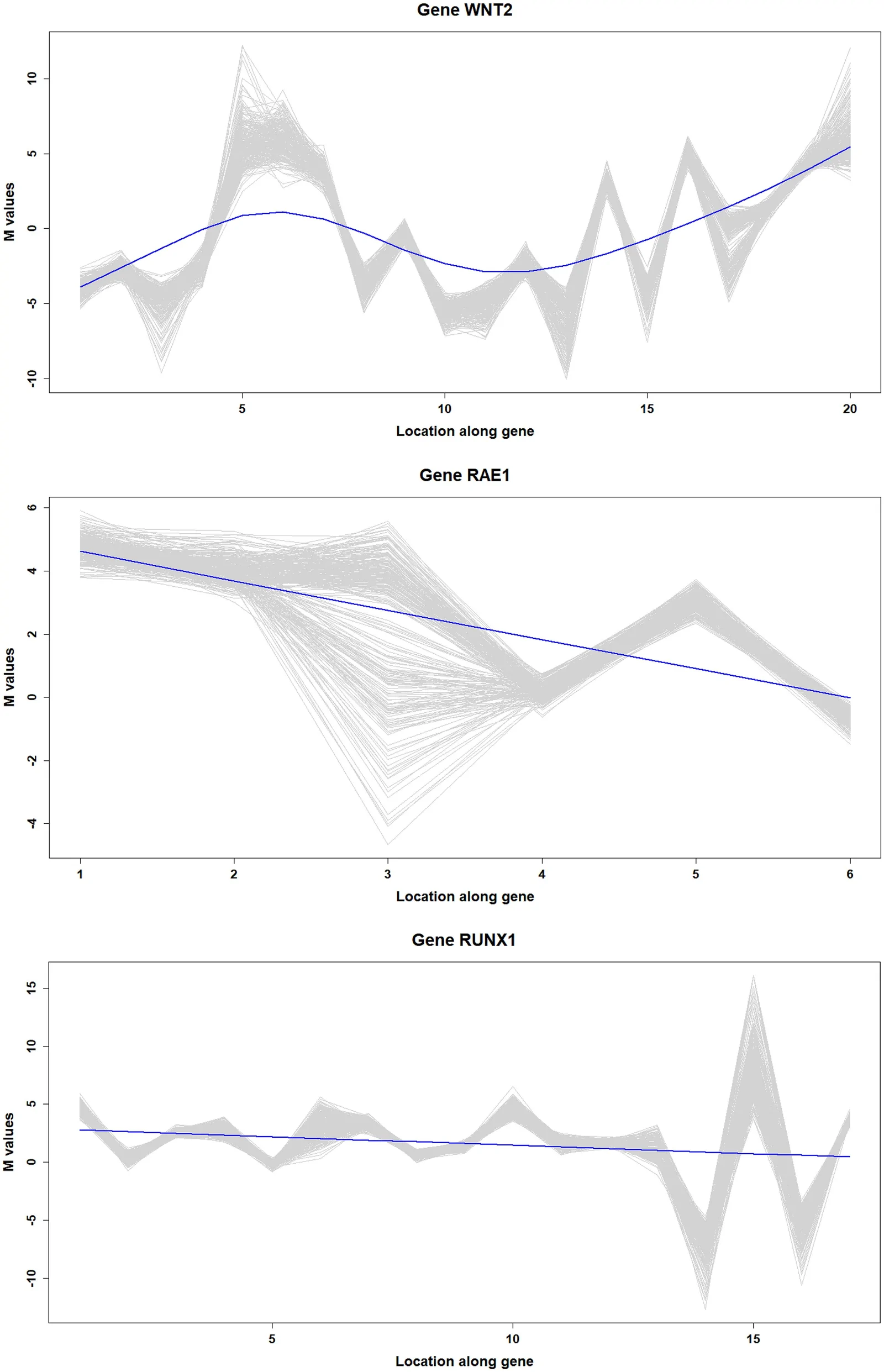
Tracts of methylation signals for the three gene regions, *WNT2, RAE1*, and *RUNX1*, identified by the gHDMA model as potential mediators. Each faint trajectory represents the methylation *M*-values across CpG sites for an individual, and the highlighted line depicts the mean functional trajectory for each gene region.

Finally, we employed a conventional site-level mediation analysis to evaluate the individual CpG sites within the gene regions identified by the gHDMA model. As shown in Fig. 9, no significant difference was observed in the mean methylation level (*β* value) of these regions between the depression case group and the healthy control group. This finding suggests that site-level mediation models may lack the statistical power to detect individual CpG sites with subtle effects on the pathway from childhood maltreatment to adult depressive symptoms. In contrast, the gHDMA model offers a key advantage: by aggregating signals across all sites within a gene region, it can amplify these minor, individually non-significant effects, thereby enabling the detection of mediation at the gene level that remains undetected by single-site approaches.

**Figure 9:**
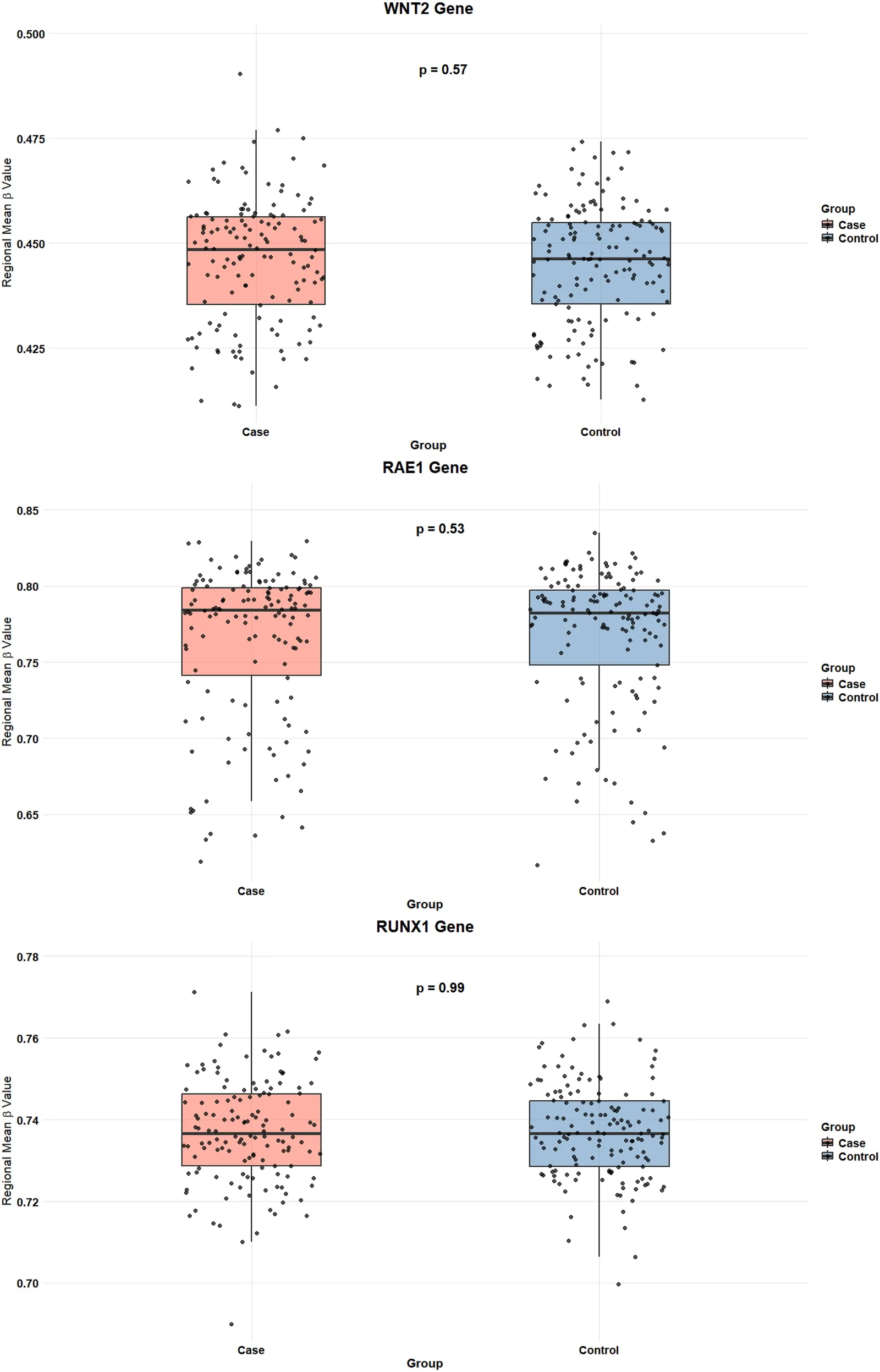
Comparison of regional mean methylation levels between depression cases and healthy controls. The boxplots with overlaid scatter points show the distribution of the mean *β* value across CpG sites within each of the three candidate gene regions (*WNT2, RAE1*, and *RUNX1*) identified by the gHDMA model. No significant differences were observed between the two groups for any of these regions (all *P>.05*).

Having identified candidate gene regions with significant mediation effects via the gHDMA model, we further conducted bioinformatic enrichment analyses to characterize the biological functions of these implicated genes. Enrichment results revealed that differentially expressed genes including *WNT2, RAE1*, and *RUNX1* were markedly enriched in neuron differentiation, midbrain dopaminergic neuron development, and T cell differentiation, alongside the *WNT* and mTOR signaling cascades (Fig. 10). These functional annotations align closely with the mechanistic roles outlined above. Collectively, these findings implicate *WNT2, RAE1*, and *RUNX1* as key mediators linking childhood maltreatment to adult depression, likely via disrupted neural plasticity, defective nucleocytoplasmic transport, and dysregulated immune-inflammatory responses.

**Figure 10:**
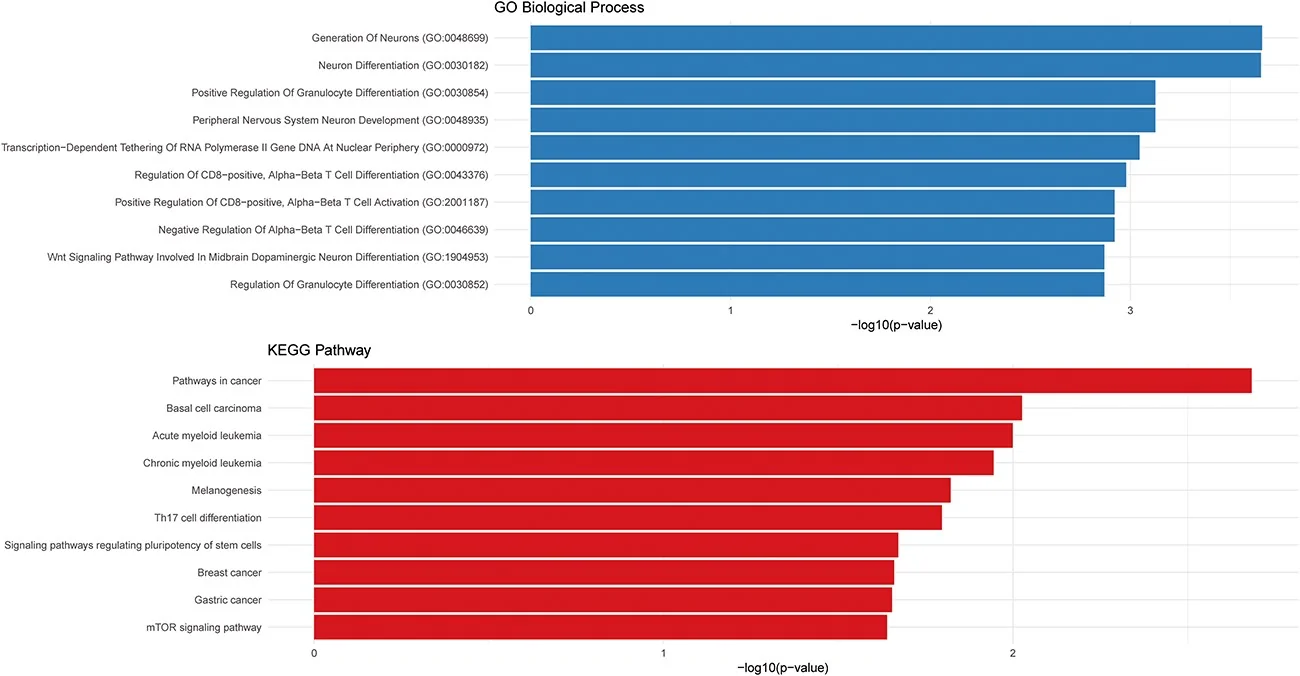
Gene Ontology (biological process) and KEGG pathway enrichment analysis for *WNT2, RAE1*, and *RUNX1* genes.

The hypothesized multi-step pathological cascade is visualized in Fig. 11. In brief, childhood maltreatment is significantly coupled with DNAm changes in *WNT2, RAE1*, and *RUNX1*. Disturbance of these genes converges to trigger disrupted neural plasticity, dysregulated immune-inflammatory responses, and impaired nucleocytoplasmic mRNA transport, collectively raising susceptibility to adult depression.

**Figure 11:**
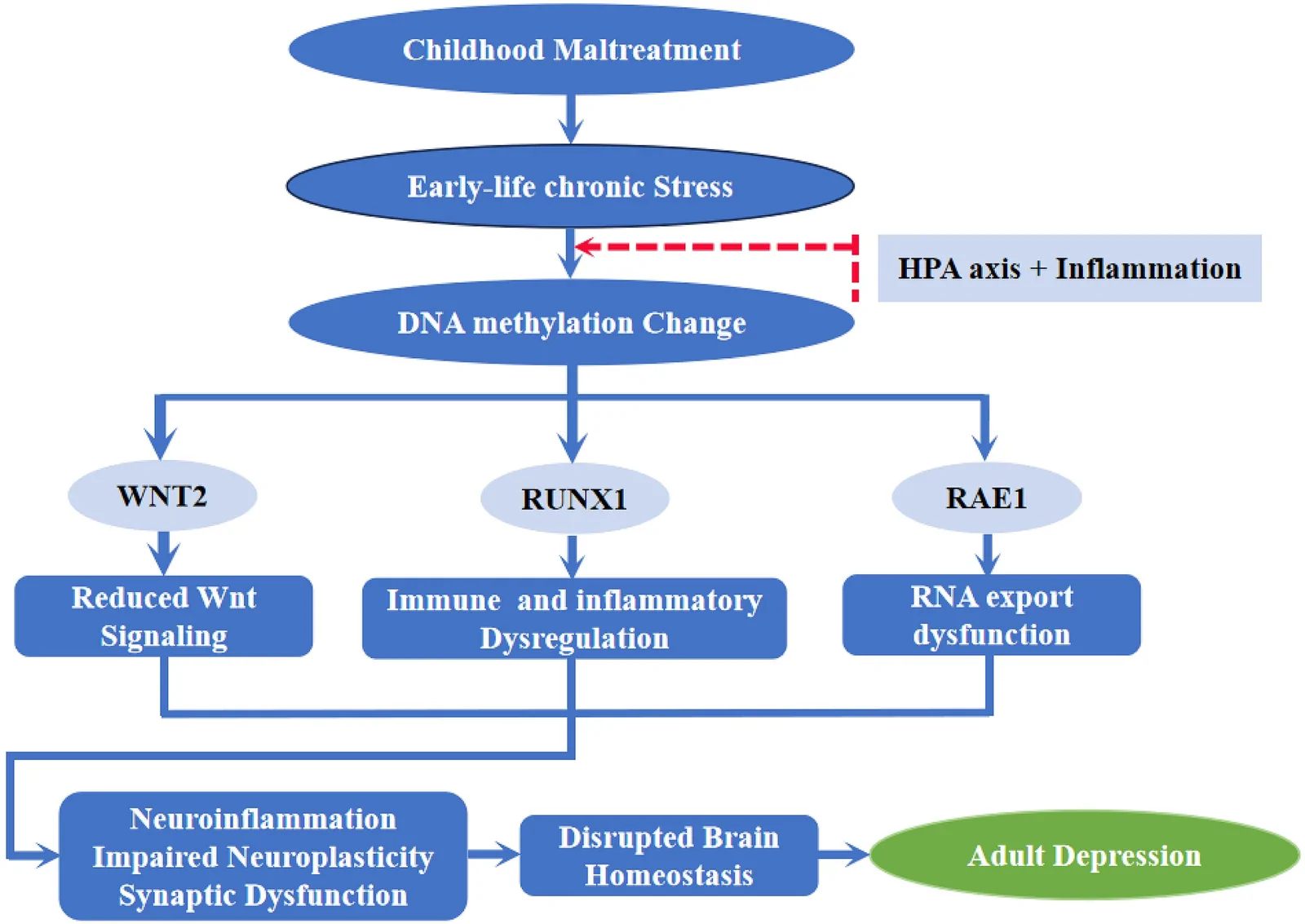
Proposed epigenetic mechanism linking childhood maltreatment to adult depression via *WNT2, RAE1*, and *RUNX1.*

## Discussion

Mediation analysis has become an important methodological tool for elucidating the mechanisms underlying complex diseases. As high-throughput omics technologies continue to advance, high-dimensional mediation analysis has increasingly turned toward identifying biologically meaningful mediators and quantifying their contributions to disease outcomes [4, 5]. From a biological standpoint, genes offer stable and interpretable functional units; gene-level analysis may therefore offer greater biological interpretability and generalizability than analyses at the level of individual CpG sites. In this study, we combined FPCA-based extraction of gene-region DNAm features, SIS screening based on Wilks’ *\varLambda*-test, de-sparsified Lasso inference, Fisher's *P*-value combination, and joint significance test to build a gene-level high-dimensional mediation analysis framework. The approach summarizes correlated methylation patterns within gene regions and substantially reduces the dimensionality entering downstream inference.

FPCA efficiently captures heterogeneous methylation patterns within gene regions, especially when CpG sites are unevenly spaced or partially missing. By retaining the dominant functional variation, it yields a small number of informative components for subsequent mediation analysis. Our simulations—covering different basis functions, dimensional settings, and both continuous and binary outcomes—showed that the proposed framework maintains satisfactory performance across a wide range of scenarios. However, FPCA can dilute weak, highly localized methylation signals that contribute little to overall regional variation. Moreover, the quality of the functional representation depends on choices of basis functions, knot locations, and smoothing parameters. While our simulations suggest limited sensitivity to these choices, more adaptive, data-driven functional representations could further improve robustness in real methylation datasets.

When applied to the GTP cohort, the framework identified gene regions whose methylation patterns may mediate the link between childhood maltreatment and adult depression. These findings offer potential molecular insight into how early-life environmental exposures shape later disease outcomes through epigenetic mechanisms. Still, the identified signals should be viewed as statistical mediation associations rather than definitive causal effects, because residual or unmeasured confounding among exposure, mediators, and outcome cannot be fully ruled out. Future work incorporating causal mediation methods, sensitivity analyses, or independent biological validation will help strengthen any causal interpretation of these results.

Several methodological limitations deserve mention. Although FPCA, SIS screening based on Wilks'*\varLambda*-test, and de-sparsified Lasso are each well-established on their own, combining them creates a hierarchical inferential procedure that extends beyond the theoretical guarantees of the original de-sparsified Lasso framework. In particular, the impact of functional truncation and screening on downstream inference has not yet been fully characterized theoretically. Our simulation studies provided empirical evidence of reasonable Type I error control and statistical power, but developing rigorous asymptotic theory for the entire pipeline remains an important avenue for future research.

Finally, the current framework treats gene regions as the primary mediation units and does not explicitly model interactions among multiple mediator genes. Such gene–gene interactions likely play important roles in complex disease mechanisms, but incorporating all pairwise interactions would cause a massive jump in dimensionality. For instance, in our GTP cohort analysis, ${n}_1=13\ 869$ mediator genes were retained after quality control, yielding ${n_1 \choose 2}=\frac{n_1\left({n}_1-1\right)}{2}\approx 9.6168\times{10}^7$ pairwise interaction terms. Designing computationally efficient strategies to accommodate gene–gene and possibly multi-omics interactions is therefore a key direction for future work. Overall, gHDMA offers a scalable, biologically interpretable framework for gene-level high-dimensional mediation analysis while pointing to several opportunities for further methodological development.

**Key Points**

- We propose a novel gene set high-dimensional mediation analysis model (gHDMA), aiming to explore DNA methylation's mediating role between environmental exposure and disease outcomes at the gene level, which addresses the limitations of single-site CpG analysis (poor biological interpretability and low statistical power).
- Integrating functional principal component analysis, SIS screening based on Wilks' *\varLambda*-test, de-sparsified Lasso, Fisher's *P*-value combination, and joint significance test, gHDMA identifies methylated genes with potential mediating effects, featuring high statistical power and robust performance.
- Compared with existing methods, gHDMA has advantages in enhanced gene-level biological interpretability, explicit modeling of CpG spatial correlation, robustness to data sparsity, and higher power in detecting weak gene-level mediating effects.

## Data Availability

The code supporting the findings of this study is publicly available at: https://github.com/YuzhaoGao/gHDMA. The datasets analyzed in this article are accessible from the Gene Expression Omnibus (GEO) repository via accession number GSE72680.
